# Effects of fear of cancer recurrence on subjective physical and mental health in breast cancer patients: The intermediary role of heart rate variability

**DOI:** 10.1002/cam4.6250

**Published:** 2023-06-16

**Authors:** Qianqian Zhang, Yinlian Cai, Sheng Yu, Lingxue Tang, Wen Li, Senbang Yao, Xucai Zheng, Jianjun Liu, Gongpu Chen, Chen Gan, Jian Xu, Huaidong Cheng

**Affiliations:** ^1^ Department of Oncology The Second Hospital of Anhui Medical University Hefei China; ^2^ Department of Head and Neck Surgery, West District of The First Afﬁliated Hospital of University of Science and Technology of China Division of Life Sciences and Medicine, University of Science and Technology of China 230601 Anhui Hefei China; ^3^ Shenzhen Clinical Medical School of Southern Medical University. Shenzhen China; ^4^ Department of Oncology Shenzhen Hospital of Southern Medical University 518000 Guangdong Shenzhen China

**Keywords:** autonomic nervous system, breast cancer, fear of recurrence, heart rate variability, psychological distress, SF‐36

## Abstract

**Background:**

Fear of cancer recurrence (FCR) and psychological distress are common psychological problems in breast cancer (BC) patients and ultimately affecting their health‐related quality of life (HRQoL). Heart rate variability (HRV) can reflect the activity of the parasympathetic nervous system. However, the pathways through which HRV influences between FCR and HRQoL are unclear. This study preliminarily explored the intermediary role of HRV in FCR and HRQoL in BC patients.

**Methods:**

A total of 101 BC patients participated in this study. HRV parameters were measured by a 5‐min dynamic electrocardiogram. FCR, psychological distress and HRQoL were evaluated by the Fear of disease progression simplified scale (FOP‐Q‐SF), Distress thermometer and SF‐36 concise health survey. The intermediary effect model was established to test the intermediary effect of high frequency‐HRV (HF‐HRV) on FCR and HRQoL.

**Results:**

FCR and psychological distress were negatively correlated with HRV in the time domain, negatively correlated with HF‐HRV in the frequency domain, and positively correlated with low frequency/high frequency (LF/HF). HF‐HRV had a partial mediating effect on the FCR and physical health and mental health, with effects of 30.23% and 9.53%, respectively.

**Conclusion:**

FCR and psychological distress are correlated with HRV parameters in the time domain and the frequency domain, and we preliminarily believe that parasympathetic nerves play an important intermediary role between FCR and subjective physical and mental health. This may provide intervention information for improving the HRQoL of BC patients.

## 1 INTRODUCTION

The incidence rate of breast cancer (BC) is increasing rapidly. It is predicted that there will be nearly 3.2 million new cases worldwide every year beginning in 2030.^1^ Although the prognosis of BC is inconsistent in different countries, the survival rate is generally on the rise.^2^ The improved prognosis is due to earlier diagnosis and progress in cancer treatment,^3^ including local treatment (surgery and radiotherapy) and systemic treatment. Systemic therapy includes neoadjuvant chemotherapy and postoperative chemotherapy, hormone receptor‐positive endocrine therapy, HER‐2‐positive targeted therapy, bone stabilization, and immunotherapy.^4^ However, during or after treatment, cancer treatment may cause different degrees of physical damage and psychological burden to patients, such as body image changes after mastectomy, toxicity and other side effects of radiotherapy and chemotherapy (such as chemotherapy/radiotherapy‐induced cognitive impairment), and negative emotions (such as anxiety, depression, and fear of cancer recurrence (FCR)).^5^, ^6^, ^7^, ^8^

FCR has become a common mood disorder leading to poorer health‐related quality of life (HRQoL).^9^, ^10^ FCR is defined by “worry or fear about the possibility of cancer recurrence or progression”.^11^ After investigating different cancer types, Moderate to high FCR were reported in 31%–49.1% of adolescent and young adult cancer survivors, and the younger the BC survivors were, the longer the duration of FCR was.^12^, ^13^ HRQoL is a key outcome measure related to mortality risk, covering physical, psychological, social, and other domains.^14^, ^15^ BC survivors' fear of cancer recurrence leads to decreased role functioning and increased psychological distress.^16^ In particular, BC survivors aged 50 or below have obvious emotional disorders related to psychosocial and hormonal changes, which can lead to increased examination behavior and even overtreatment, this can have adverse effects on the patients' physical and mental health.^17^, ^18^ Therefore, by assessing the degree of FCR as early as possible and intervening, the HRQoL and survival time of patients can be improved.

The autonomic nervous system (ANS) includes the parasympathetic and sympathetic nerves. The activities of the ANS are related to emotional disorders.^19^ At present, evaluating the regulation of autonomic nervous system is achieved through non‐invasive measurement of heart rate variability (HRV). A decrease in HRV is a sign of autonomic dysfunction and poor health.^20^ Long‐term chronic psychological stress overactivates the sympathetic nervous system and inhibits the parasympathetic nerve.^21^ HRV is also related to cognition, emotion, and social function.^22^ At present, few studies have explored the relationship between FCR, psychological distress, HRV and HRQoL (physical health and mental health). The impact pathway of HRV between FCR and HRQoL is unclear. If HRV does play a mediating role, then interventions to improve HRV could be used to improve HRQoL in BC patients. Therefore, this study explored (1) the differences in FCR, psychological distress and HRQoL between a high HRV group and a low HRV group; (2) the correlation between FCR, psychological distress, quality of life and HRV parameters; and (3) whether high frequency‐heart rate variability (HF‐HRV) can predict HRQoL (physical health and mental health) and whether HF‐HRV plays an intermediary role between FCR and HRQoL of BC patients. Our findings will provide valuable information for interventions related to the improvement of HRQoL in BC patients.

## 2 MATERIALS AND METHODS

### 2.1 Participants

A total of 101 female BC patients were enrolled in this study from the Second Affiliated Hospital of Anhui Medical University (37 cases) and Anhui Cancer Hospital (64 cases) from March 2022 to October 2022. This study was approved by the Ethics Review Committee of the Second Affiliated Hospital of Anhui Medical University and the Cancer Hospital. All subjects (101 patients) provided written informed consent. Our research is conducted in accordance with the Helsinki Declaration.

Inclusion criteria: (1) pathological diagnosis (2) no consumption of energy drinks (coffee, tea, alcohol) or smoking 3 h before the assessment; (3) no lactation or pregnancy; and (4) no communication barriers that would impede understanding contents of the questionnaire. Exclusion criteria: (1) history of heart disease such as arrhythmia and hypertension, evidence of liver, or kidney failure and hyperthyroidism; (2) more than 5 h of strenuous exercise per week; (3) presence of mental disorders or inability to accurately express inner thoughts; and (4) advanced cachexia. Sociodemographic and clinical data were collected through hospital electronic cases.

### 2.2 Questionnaires

#### 2.2.1 Distress thermometer

Psychological distress was a visual analog scale ranging from 0 (no psychological distress at all) to 10 (extreme psychological distress), with a score of ≥4 indicating clinically meaningful distress, and a higher score indicated a higher degree of psychological distress.^23^

#### 2.2.2 Fear of cancer recurrence

The fear of progression questionnaire‐short form for cancer patients (FOP‐Q‐SF) is a one‐dimensional simplified scale verified by 2006 by Mehner^24^ in Germany; the scale is self‐rated by BC patients. The scale has 12 items in total and adopts a Likert 5‐grade score. A higher score corresponds with a higher degree of FCR.

#### 2.2.3 Health‐related quality of life

HRQoL is surveyed through SF‐36 (Chinese version 1.0). The SF‐36 is a self‐reported health measurement method that has good internal consistency and moderately good structural validity for BC patients.^25^ It contains 36 questions, 8 dimensions, and 2 fields. Physical functioning (PF), bodily pain (BP), role‐physical (RP) and general health (GH), are the four dimensions that constitute the physical component scale (PCS); vitality (VT), role‐emotional (RE), social functioning (SF) and mental health (MH), are the four dimensions that make up the mental component scale (MCS). Each score is standardized from 0 to 100, and lower scores correspond with worse health.

#### 2.2.4 ECG collection

We used a HeaLink R211B microelectrocardiogram (ECG) recorder (Healink Ltd.) to measure the 10‐min supine ECG signal of BC patients in the ward. The collection rate of the ECG recorder is 400 Hz, and its signal bandwidth is 0.6–40 Hz. After completing the questionnaire, physiological measurements were taken. Before starting the signal recording, we confirmed that the patient did not take caffeine or tobacco within 3 h prior to the assessment, and the patient was instructed to lie down and be quiet for 10 min before the ECG acquisition. During the measurement, patients were required to continue lying down, not talk or move, and breathe naturally.

#### 2.2.5 Heart rate variability analysis

We checked the signal quality for any abnormalities. If there was an abnormality, the case was removed. Then, based on the Pan‐Tompkins algorithm,^26^ we extracted the ECG R‐R Beat‐to‐Beat Interval Time Series. The data were transferred to the Kubios HRV software (version 3.5.0).^27^ We then checked the movement of the ECG signal and selected segments with no or minimal movement for 5 min for each participant. The standard algorithm implemented in Kubios software was used to analyze the HRV data of 5 min. HRV parameters in the time domain included (i) SDNN (ms), the standard deviation of all normal‐to‐normal cardiac intervals; (ii) RMSSD (ms), the square root of the mean of the sum of the squares of differences between adjacent normal‐to‐normal cardiac intervals characterized by frequency domain fast Fourier transform (FFT), normalized HF‐HRV (0.15–0.40 Hz) power and ratio of low frequency to high frequency (LF/HF). Based on a meta‐analysis, this analysis detected the critical values of SDNN in low HRV and high HRV cancer patients, we used a cutoff value of SDNN<20 ms as low HRV,^28^ and we divided the study participants into groups; patients with an SDNN<20 ms were included in the low HRV group, and patients with an SDNN≥ 20 ms were included in the high HRV group.

#### 2.2.6 Statistical analysis

We used IBM SPSS statistical software (version 25.0) to analyze the data. First, we used the Shapiro–Wilk normality test to test the normality of the data. Due to the nonnormality, the categorical variables were analyzed by the chi‐square test for the comparison of demographic data, and quantitative variables were analyzed by the Mann–Whitney *U* test to compare psychological distress, FCR and SF‐36 scores for both the high and low HRV groups. We use Spearman rank correlation to analyze the correlation size of each variable.

To test the correlation between HF‐HRV and quality of life, we used resting HF‐HRV as a predictor of HRQoL (including physical health and mental health) for multiple linear regression. Although there was no significant difference in some factors (such as age, BMI, KPS, and tumor stage), they were controlled according to previous studies.

In this study, the mediation effect of HF‐HRV on FCR and HRQoL was evaluated using Model 4 of the PROCESS v4.0 plugin developed by Hayes (2013).^29^ The 95% confidence interval was calculated using the bootstrap method with a sampling frequency of 5000 times. For the significance test, the bootstrap method (with 5000 samples) was better than the Sobel test.^30^

## 3 RESULTS

### 3.1 Demographic data of the participants

The demographic and treatment‐related characteristics of our participants who collected ECG data are shown in Table 1. 101 BC patients were included after excluding the data of 3 patients with ECG deficiency. Patients with SDNN<20 ms were enrolled in the low HRV group (64 cases), and patients with SDNN≥20 ms(37 cases) were enrolled in the high HRV group, aged 53 (47.25,56) versus 51 (39.50,54), respectively. The BMI was 23.88(21.48, 26.04) versus 23.44(21.91, 25.76), and 45.3%(29) versus 48.6% (18) patients in the second stage, with 65.3% and 52.5% patients receiving chemotherapy and targeted therapy alone or in combination, respectively. This demographic information has no statistical significance.

**TABLE 1:** Baseline data of the patients in the High SDNN (≥20 ms) group and Low SDNN (<20 ms)group.

Characteristics	Total (*n* = 101) ¯M(*P*_*25*_, *P*_*75*_)	Low SDNN(*n* = 64) ¯ M(*P*_*25*_, *P*_*75*_)	High SDNN (*n* = 37) ¯ M(*P*_*25*_, *P*_*75*_)	*p‐*value
Age (years)	53 (44,56)	53 (47.25,56)	51 (39.50,54)	0.08
BMI (kg/m^2^)	23.83 (21.54,26)	23.88 (21.48,26.04)	23.44 (21.91,25.76)	0.93
KPS	80 (70,80)	80 (70,80)	80 (70,80)	0.10
	Total (*n* = 101) *n* (%)	Low SDNN (*n* = 64) *n* (%)	High SDNN (*n* = 37) *n* (%)	*p‐*value
BMI classification				0.79
Normal weight (18.5 > 24.9 kg/m2)	63.4 (63.4)	40 (62.5)	24 (64.9)	
Overweight (25 > 29.9 kg/m2)	33 (32.7)	34.4 (22)	29.7 (11)	
Obese (≥ 30 kg/m2)	4 (4)	2 (3.1)	2 (5.4)	
Employment status				0.38
Unemployed	62 (61.4)	37 (57.8)	25 (67.6)	
Employed	32 (31.7)	21 (32.8)	11 (29.7)	
Retired	7 (6.9)	6 (9.4)	1 (2.7)	
Education				0.25
illiteracy	17 (16.8)	14 (21.9)	3 (8.1)	
Primary school	24 (23.8)	15 (23.4)	9 (24.3)	
Middle school	42 (41.6)	26 (40.6)	16 (43.2)	
University and above	18 (17.8)	9 (14.1)	9 (24.3)	
Breast surgery type				0.86
Mastectomy	84 (83.2)	53 (82.8)	31 (83.8)	
Lumpectomy	7 (6.9)	4 (6.3)	3 (8.1)	
No surgery	10 (9.9)	7 (10.9)	3 (8.1)	
Disease stage				0.70
I	17 (16.8)	11 (17.2)	6 (16.2)	
II	47 (46.5)	29 (45.3)	18 (48.6)	
III	19 (18.8)	14 (21.9)	5 (13.5)	
IV	18 (17.8)	10 (15.6)	8 (21.6)	
Current cancer treatments received				
Chemotherapy	66 (65.3)	41 (64.1)	25 (67.6)	0.72
Radiotherapy	4 (4)	2 (3.1)	2 (5.4)	0.97
Targeted therapy	53 (52.5)	35 (54.7)	18 (48.6)	0.56
Endocrine therapy	5 (5)	3 (4.7)	2 (5.4)	1.00
HER‐2				0.34
−	50 (49.5)	34 (53.1)	16 (43.2)	
+	51 (50.5)	30 (46.9)	21 (56.8)	

*Note*: The data are expressed as median (upper and lower quartiles) M (*P25*, *P75*) or *n* (%); Low SDNN represents the low heart rate variability group; High SDNN represents the high heart rate variability group.

Abbreviations: BMI, body mass index; KPS, Karnofsky performance status.

### 3.2 Differences in FCR, psychological distress, and HRQoL between the low and high HRV groups

Compared with those of patients in the low HRV group (SDNN<20 ms), the FCR and psychological distress scores of patients in the high HRV group were lower (Table 2, 35 (25, 44) versus 40 (32, 47), 4 (3, 6) versus 7 (4, 8); *z* = −2.388, *z* = −3.928; *p* = 0.017, *p* < 0.001, respectively). BC patients with low HRV had lower subscales scores for the SF‐36 but no significant differences in SF. The scores of PCS and MCS in low HRV group were lower compared with high HRV group (57.3 (50,65), 71.3 (61,77), 46.6 (34,58), and 62.9 (39,76), respectively). *z* = −4.156，*z* = −2.823； *p* < 0.001, *p* = 0.005, respectively.

**TABLE 2:** Low SDNN is associated with high FCR, high psychological distress and poor HRQoL.

	Low SDNN (*n* = 64) ¯M(*P*_*25*_, *P*_*75*_)	High SDNN (*n* = 37) ¯M(*P*_*25*_, *P*_*75*_)	*Z*	*p*
FCR	40 (32,47)	35 (25,44)	−2.388	0.017
Psychological distress	7 (4,8)	4 (3,6)	−3.928	<0.001
PF	65 (55,75)	75 (70,80)	−4.747	<0.001
RP	50 (50,75)	75 (75,75)	−4.050	<0.001
BP	72 (52,84)	84 (54,90)	−2.129	0.033
Gh	45 (35,50)	55 (40,68)	−2.563	0.010
VT	50 (35,63)	55 (45,75)	−2.529	0.011
SF	50 (37,75)	75 (37,87)	−1.930	0.054
RE	33.3 (33,66)	66.7(33,66)	−2.576	0.010
MH	42 (24,56)	60 (36,72)	−2.950	0.003
PCS	57.3 (50,65)	71.3(61,77)	−4.156	<0.001
MCS	46.6 (34,58)	62.9 (39,76)	−2.823	0.005

*Note*: Low SDNN is compared with High SDNN. The data are represented by the median (upper and lower quartiles) M (*P25, P75*).

Abbreviation: BP, bodily pain; FCR, fear of cancer recurrence; GH, general health; HRQoL: health‐related quality of life; MCS, mental health status; MH, mental health; PCS, physical health status; PF, physical functioning; RE, role‐emotional; RP, role‐physical; SF, social functioning; VT, vitality.

### 3.3 Correlation between HRV index, FCR, psychological distress, and HRQoL in patients with BC

Spearman results showed that HF‐HRV was correlated with FCR and psychological distress (*r* = −0.538, *r* = −0.760, *p* < 0.001, respectively), and HF‐HRV has a positive correlation with PCS and MCS (*r* = 0.471, *r* = 0.590, *p* < 0.001, respectively) (Table 3). The correlations between FCR, psychological distress, and each parameter of HRV are presented in Table 3.

**TABLE 3:** Correlation between heart rate variability with FCR and psychological distress and HRQoL in BC patients.

	RMSSD	SDNN	HF‐HRV	LFHF	PF	RP	BP	Gh	VT	SF	RE	MH	PCS	MCS	FCR
SDNN	0.961**														
HF‐HRV	0.618**	0.490**													
LFHF	−0.619**	−0.491**	−1.000**												
PF	0.400**	0.431**	0.347**	−0.345**											
RP	0.463**	0.454**	0.391**	−0.390**	0.718**										
BP	0.252*	0.247*	0.351**	−0.351**	0.423**	0.363**									
Gh	0.258**	0.231*	0.401**	−0.399**	0.495**	0.558**	0.460**								
VT	0.246*	0.244*	0.360**	−0.358**	0.377**	0.410**	0.480**	0.598**							
SF	0.290**	0.260**	0.606**	−0.605**	0.301**	0.315**	0.478**	0.536**	0.615**						
RE	0.337**	0.256**	0.617**	−0.616**	0.404**	0.392**	0.546**	0.555**	0.544**	0.709**					
MH	0.348**	0.293**	0.483**	−0.483**	0.321**	0.397**	0.511**	0.613**	0.721**	0.711**	0.722**				
PCS	0.429**	0.423**	0.471**	−0.469**	0.776**	0.792**	0.761**	0.792**	0.621**	0.546**	0.629**	0.619**			
MCS	0.355**	0.311**	0.590**	−0.590**	0.393**	0.433**	0.561**	0.660**	0.824**	0.888**	0.835**	0.905**	0.684**		
FCR	−0.329**	−0.286**	−0.538**	0.538**	−0.344**	−0.404**	−0.602**	−0.603**	−0.734**	−0.741**	−0.781**	−0.813**	−0.650**	−0.876**	
Psycho‐logical distress	−0.503**	−0.433**	−0.760**	0.761**	−0.364**	−0.456**	−0.523**	−0.553**	−0.582**	−0.803**	−0.798**	−0.802**	−0.631**	−0.858**	0.807**

* *p* < 0 0.05

** *p* < 0.01.

### 3.4 Resting HF‐HRV and HRQoL (including Physical and Mental Health)

To test the relationship between HF‐HRV and HRQoL, we conducted a multiple linear regression. After including demographic covariates and covariates related to HF‐HRV, the results of the multiple linear regression (Table 4) showed a significant correlation between PCS, MCS, and HF‐HRV. (*b* = 0.238, SE = 0.043, *p* < 0.001, 95% CI [0.15, 0.33]), (*b* = 0.429, *p* < 0.001, SE = 0.065, 95% CI [0.30, 0.56]) Therefore, higher HF‐HRV has predictive value for the HRQoL of BC patients.

**TABLE 4:** Multivariate linear regression for predictive factors on PCS and MCS (*n* = 101).

Variables entered	PCS				MCS			
	β(SE)	*b*	*p*	*F*	β (SE)	*b*	*p*	*F*
Age (years)	−0.085 (0.093)	−0.120	0.201	33.492***	−0.060 (0.139)	−0.106	0.446	17.707***
BMI (kg/m^2^)	−0.148 (0.272)	−0.618	0.025		0.039 (0.406)	0.203	0.617	
KPS	0.609 (0.110)	1.039	<0.001		0.345 (0.164)	0.736	<0.001	
Disease stage	−0.069 (0.876)	−0.968	0.272		−0.057 (1.308)	−0.998	0.447	
HF‐HRV	0.355 (0.043)	0.238	<0.001		0.510 (0.065)	0.429	<0.001	
R^2^	0.638				0.482			

Abbreviations: β, regression coefficient, b, standardized regression coefficient, SE = standard error.

*Note*: Adjusted for age, BMI, KPS, Tumor stage. A lack of punctuation of “*” indicates that *p*‐value is no significant difference.

*** *p* < 0.001.

### 3.5 Test the Mediation Model with FCR as the Independent Variable

**FIGURE 1:**
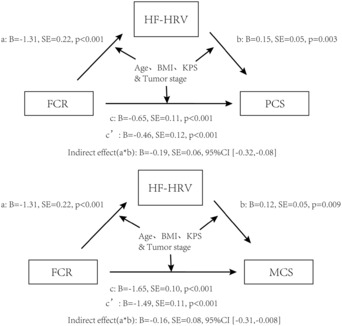
Controlling for age and BMI, KPS, and tumor stage in all mediating models, FCR as the dependent variable, and examining the indirect effects of HF‐HRV on PCS and MCS, respectively. Path a represents the relationship between FCR and HF‐HRV. Path b denotes the relationship between HF‐HRV and PCS and MCS, respectively. Path c is the total effect of FCR on PCS and MCS, and Path c’ is the direct effect of FCR on PCS and MCS after controlling HF‐HRV. In these two models, the difference between the total effect and the direct effect or indirect effect is significant, indicating that HF‐HRV is an important medium.

We next tested whether the association between FCR and physical and mental health was mediated by HF‐HRV. These models are shown in Figure 1. After controlling for age, BMI, KPS, and tumor stage, FCR was used as the independent variable, HF‐HRV was used as the intermediate variable, and physical and mental health in SF‐36 was used as the dependent variable, our results suggest that HF‐HRV has a partly intermediate effect between FCR and physical health, which accounted for 30.23% of the total effect. HF‐HRV partially mediated FCR and mental health effects, accounting for 9.53% of the total effects.

## 4 DISCUSSION

Our results showed that FCR and psychological distress were negatively correlated with SDNN and RMSSD; additionally, they were positively correlated with LF/HF. The low SDNNs (< 20 ms) had higher scores of psychological distress and FCR and poorer HRQoL. HRV can predict the physical and mental health of BC patients. The FCR and HRQoL of BC patients may be mediated by HF‐HRV.

SDNN, RMSSD, and HF‐HRV represent vagal activity, while the LF/HF ratio reflects sympathetic nerve balance or sympathetic regulation.^31^ We found that psychological distress was negatively correlated with RMSSD, SDNN, and HF‐HRV. A study suggests that higher parasympathetic nerve activation (as an indicator of higher HRV) was associated with fewer physical symptoms.^32^ Another study showed that compared with the healthy control group, pathological apprehensores had less sympathetic nerve excitement and parasympathetic nerve activation when they were exposed to fear.^33^ This result indicates that fear is related to LF/HF. Multiple studies have shown that lower HRV is related to psychological disorders, including anxiety, depression and other mental disorders.^34^, ^35^, ^36^ Consistent with other studies, our findings indicate that psychological distress and FCR are negatively correlated with vagus nerve activity. However, in a study of stage I (66%) and stage II (10%) BC, HF‐HRV was detected during radiotherapy and 10 days after radiotherapy, and it was found that HF‐HRV was not related to FCR.^37^ These findings suggested that ionizing radiation can interfere with the heart and affect the results of HF‐HRV testing during radiotherapy for BC.^38^ This may be the reason why this study was inconsistent with other studies.

Lower HRV is a predictor of disease and adverse events in patients.^39^ This is consistent with our research results. After controlling for age, BMI, KPS and tumor stage, low HF‐HRV predicted poor HRQoL in BC patients. Recently, it has been found that ANS in tumors is usually associated with angiogenesis and immunosuppression in the tumor microenvironment, thus participating in the development and progression of cancer.^40^ An animal experiment showed that there are nerve fibers embedded in BC masses, and the ANS has a direct influence on tumor growth and metastasis. In addition, it has been confirmed that there is a direct neural connection between the brain and breast tumors through the vagus nerve.^41^ Increasing evidence has shown that alpha‐adrenergic receptors were associated with the growth and metastasis of breast cancer cells.^42^ In addition, research has shown that higher FCR is associated with lower quality of life. Healthy behaviors, psychological reactions and dysfunction were identified as consequences of FCR.^43^ Our research also showed that BC patients with a higher FCR have worse physical and mental health. There is currently limited research on FCR, HRV, and HRQoL in BC patients. Our results indicate that FCR affects patients' physical health. FCR may affect the health of BC patients through HF‐HRV. Patients with low parasympathetic activity and high FCR showed poor physical health. Therefore, we speculate that HF‐HRV mediates the relationship between FCR and physical health in BC patients.

Our results showed that HRV predicts mental health in patients with BC. A meta‐analysis showed that HRV was significantly correlated with cerebral blood flow in the ventral prefrontal cortex and the amygdala. Regardless of age, the resting HRV (RMSSD) was highly correlated with the functional connectivity of the medial prefrontal cortex and the amygdala, which has been associated with cerebral mechanisms of emotional regulation.^44^ Research shows that the amygdala is associated with conditioned fear and anxiety,^45^ and patients with higher HRV have better emotional expression.^46^ Consistent with our findings, patients with low HRV also had reduced mental health and emotional functioning. Notably, HF‐HRV still predicts mental health in BC patients after controlling for covariates (age, BMI, KPS, tumor stage), and FCR further aggravates the emotional function of the patient. Patients with low parasympathetic activity and high FCR showed poor mental health given the short‐term HRV of the HF‐HRV frequency domain analysis response.^47^

## 5 LIMITATIONS

Our study had some limitations. This preliminary study has a small sample size; therefore, our findings and explanations should be confirmed by further clinical exploration with larger sample sizes. Although this study found that high FCR and low HF‐HRV predicted a decline in HRQoL, it could not explain the causal relationship between these factors. Further research on this relationship requires longitudinal research. Given the above limitations, our model results should be considered preliminary. In addition, the interbeat changes in the RR interval time series are affected by the ANS and the patient's breathing. In future research, ECG‐derived breathing technology should be considered for this measurement.

## 6 CONCLUSION

The FCR is correlated with HRV time and frequency domain parameters. BC patients with high levels of FCR and downregulation of parasympathetic activity also had poor HRQoL. We preliminarily believe that parasympathetic nerves may play an important intermediary role between FCR and HRQoL. Our research on the connection between HRV, FCR and psychological distress in BC patients may improve our understanding of the role of the ANS. This requires further measurement of the dynamic processes of FCR and HRV to determine the mediating effect of HF‐HRV, which will provide accurate intervention information for improving the physical health and mental health of cancer patients.

## AUTHOR CONTRIBUTIONS

**Qianqian Zhang:** Writing – original draft (equal); writing – review and editing (equal). **Yinlian Cai:** Investigation (equal). **Sheng Yu:** Investigation (equal). **Lingxue Tang:** Data curation (supporting). **Wen Li:** Data curation (supporting); writing – review and editing (supporting). **Senbang Yao:** Data curation (supporting). **Xucai Zheng:** Data curation (supporting). **Jianjun Liu:** Resources (supporting). **Gongpu Cheng:** Resources (supporting). **Chen Gan:** Resources (supporting). **Jian Xu:** Data curation (supporting). **Huaidong Cheng:** Validation (supporting); writing – review and editing (supporting).

## FUNDING INFORMATION

This study was supported by the National Natural Science Foundation of China (No. 81872504).The funders had no role in the study design, data collection and analysis, decision to publish, or preparation of the manuscript.

## CONFLICT OF INTEREST STATEMENT

The authors declare that the research was conducted in the absence of any commercial or financial relationships that could be construed as a potential conflict of interest. All authors declare that they have no conflicts of interest for this study.

## ETHICS STATEMENT

Studies involving human participants were approved by the Second Affiliated Hospital of Anhui Medical University and the Ethics Review Committee of Anhui Oncology Hospital (2012088). Written informed consent was provided by the patient/participant to participate in the study.

## Data Availability

Availability of data and materialsAll data required to evaluate the paper conclusions are presented in the paper and/or attached file.
